# Gynaecological Malignancies from Palliative Care Perspective

**DOI:** 10.4103/0973-1075.76243

**Published:** 2011-01

**Authors:** Kamlesh Mishra

**Affiliations:** Consultant Gynecology Oncology, Dharamshila Cancer Hospital & Research Centre, New Delhi, India

**Keywords:** Gynecological malignancies, Palliative care, Symptomatic care

## Abstract

Of the approximately 80,000 new cases of all cancers detected every year in India, 10–15% are gynecological malignancies. As per population-based registries under the National Cancer Registry Program, the leading sites of cancer among women are the cervix uteri, breast, and oral cavity. About 50–60% of all cancers among women in India are mainly of the following four organs: cervix uteri, breast, corpus uteri, and ovaries. Over 70% of these women report for diagnostic and treatment services at an advanced stage of disease, resulting in poor survival and high mortality rates. Among all gynecological cancers, ovarian cancer is the deadliest one and, in 2/3^rd^ of the cases, is detected in an advanced stage. But, in India and in other developing countries, due to inadequate screening facilities for the preventable cancer cervix, this kills more women than any other cancer in females. Gynecology Oncologist as a sub-specialist has an immensely important role in curtailing the menace of gynecological malignancies by providing comprehensive preventive, curative, palliative and follow-up services, with the aim of assuring a good quality of life to women as a cornerstone of cancer management.

## PALLIATIVE CARE IN THE PERSPECTIVE OF GYNECOLOGICAL MALIGNANCIES

Cancers of the cervix, ovary, body of uterus, vulva, persistent gestational trophoblastic tumor (GTN), vagina, and fallopian tube come under the heading of gynecological malignancies. Palliative surgical and non-surgical interventions are generally required in the setting of incurable advanced or recurrent gynecological malignancies but, sometimes even patient cured of disease may require interventions for problems developed as a result of the side-effects of curative treatment. Advanced ovarian cancer runs a chronic course; after variable phases of quiescence, it tends to recur. After the second or third or fourth line of chemotherapy and so on, a time comes when these patients are left with no available cancer specific treatment options and continue to develop progressive disease. Then, only symptomatic or palliative treatment can be offered. Similarly, many of the cancer cervix patients present at such an advanced stage that only palliative symptomatic treatment can be offered to them, while other groups may require it as a result of failure of curative attempt and to treat late recurrences. Most cases of cancer vulva are histopathologically squamous cell carcinomas and should be detected in an early stage due to its obvious location, but, unfortunately, it is not true in developing countries due to negligence on the part of the women herself and, sometimes, the treating physician tends to misinterpret this as skin infection or some other benign skin condition. Hence, diagnosis with biopsy is delayed till it becomes very advanced or metastatic. Even after diagnosis, many cases undergo suboptimal surgery. These cases later on present with fungating and ulcerating groin nodes. Lymphedema develops as a sequelae of successful treatment of cancer vulva due to inguino-femoral lymphadenectomy. Persistent GTN cases generally run an acute aggressive course and require different supportive measures, like blood replacement, control of vaginal bleeding, or brain irradiation. But, it has a good prognosis if detected on time and if the right combination of chemotherapy at the right doses is given. The following are the common symptoms of gynecological malignancies that need symptom management[1]:

## VAGINAL BLEEDING

Abnormal vaginal bleeding is the most common presenting symptom of cervical, endometrial, and vaginal cancers. In developing countries, a woman of cervical cancer presenting at an advanced stage with a huge growth replacing the whole of the cervix and with excessive bleeding is very common. Rarely, advanced cases of ovarian malignancy, if it involves the uterus and vagina, could have troublesome bleeding. In cancer cervix cases, till the diagnosis of cancer is confirmed and cancer-specific treatment could be started, all possible attempts should be made to control the vaginal bleeding. But, in the setting of progressive or recurrent disease with limited anti-cancer treatment available, a judicious decision in selecting appropriate treatment is a must. If there is distant metastasis with very short life expectancy, aggressive surgical measures like internal iliac ligation may not be justified and simple measures like vaginal packing or hemostatic injections or blood replacement etc. should be tried.

The options available to control bleeding to be tried are described in detail below:

1. Tight vaginal packing with simple roller gauge should be carried out to control bleeding. The packing to be effective to control bleeding should be done in a lithotomy position, with the speculum exposing the upper vagina, under aseptic conditions. Rarely, short general anesthesia or sedation may be required. All fornices should be packed tightly in a layered fashion till introitous, maintaining the pressure throughout. While, the pack is *in situ*, the patient should be on continuous catheterization as she may have difficulty in passing urine due to compression on the urethra. Other simple measures like restricting patient mobility or foot end elevation or hemostatic agents (local or oral or parenteral) could enhance the effectiveness of the vaginal packing. Monsel solution (ferric subsulfate) is a good hemostatic solution to be applied locally directly on the bleeding area on the cervix or with a roller gauge pack soaked in it. Even formalin solution applied on its tip enhances the effectiveness of the packing. While the patient has a pack in the vagina, it is better to give broad-spectrum antibiotics and metronidazole to check the infection. Low-grade bleeding can be controlled with intravenous Trenaxamic acid in a dose of 10 mg/kg three to four times per day. Besides measures to control bleeding, adequate blood volume should be replaced with packed red blood cells or plasma expanders.
2. Hemostatic radiation: After confirmation of histopathological diagnosis of cancer, if radiotherapy (RT) is indicated, as in cancer cervix or vagina, RT could be started in hemostatic doses. Standard fractions of RT are in 1.8–2.0 Gy in single fraction, but in the setting of palliative care or in hemostatic purposes, RT can be given in enhanced doses of 30 Gy in 10 fractions.
3. If simple measures like packing fail to control bleeding, and hemostatic RT is not possible or indicated, then the next possible option is decreasing the pulse pressure of the vessels supplying the tumor. This can be done by uterine artery embolization or surgical interventions like uterine or hypogastric arterial ligation. But, such interventions in a palliative setting in an advanced progressive disease with widespread dissemination causing imminent threat to the patient’s life are of limited value and, therefore, appropriate patient selection for meaningful benefit is a must. If excessive bleeding in an end-of-life situation occurs, only sedation to the patient to relieve discomfort and care to the relatives is important.

## VAGINAL DISCHARGE

Large cervical or vaginal growths at the time of primary presentation or recurrence can be a necrotic infected mass in the vagina producing continuously trickling of foul smelling vaginal discharge. Sometimes, pelvic recurrences of ovarian cancer can erode the vagina and produce continuous malodor. Regular Seitz bath in warm water with antiseptic solution could be of help. Systemic antibiotics and metronidazole could help in reducing the odor and infection, but in cases who have already received RT and are now suffering with progressive disease, it has limited efficacy as the vascularity in the tissues is hampered. In contrast, in cases of ovarian cancer where RT has not been received, tumor regression may be tried with local RT. Local excoriation of vulvar skin could be prevented by application of local emollients like Vaseline or petroleum jelly.

## OBSTRUCTIVE UROPATHY

Primary large cervical growth, residual/recurrent progressive disease, or less commonly post-radiation/surgical fibrosis with no recurrence may be responsible for obstructive uropathy. It is not uncommon in developing countries, for a patient of cervical cancer, to present primarily with signs and symptoms of uremia. In a study on urinary diversion in cancers, about 39% of the cases were of cervical cancer, and 36% of them presented primarily with obstructive uropathy with raised creatinine.[2] Temporary diversions with percutaneous nephrostomies (PCNs) have a definite role in primary cancers to relieve uremia, to allow curative or palliative RT to be started. But, the role of PCNs is questionable in residual or recurrent progressive disease, when no cancer-specific treatment could be offered.[3] A judicious decision and careful selection of patient is must for performing PCN. Retrograde ureteric stenting is an alterative procedure. Relatives or patient should be counselled in a proper manner and should be prompted in proper decision making, with full explanation of the pros and cons of such procedure. Sometimes, when women have recurrent progressive disease with no cancer-specific treatment available to arrest its progress, it may be wiser to allow the patient to die silently due to uremia than to aggravate her agony by allowing local progress invading the bladder or rectum, resulting in fistulae. Actually, this may enhance the patient’s misery further rather than palliating the symptoms. The end result is her forced social outcast for the rest of her life, deteriorating her quality of life to an extreme low level.

## LYMPHEDEMA

Causes of disabling edema in the lower extremity may be large tumor obstructing vein/lymphatics, deep vein thrombosis, or as a part of generalized edema with hypoproteinemia. Symptomatic relief of edema and leg discomfort is achieved with graded compression stockings, elevation of the extremities, and administration of diuretics. Around 30% of the gynecological malignancy cases develop lower limb lymphedema, which increases up to 47% in cases of inguinofemoral lymph node dissection, followed by RT in vulvar malignancies.[4] In a study at Sloan Kattering Hospital, in 1280 patients of uterine corpus cancer, 5.7% developed lower limb lymphedema following treatment.[5] The authors found an association between pelvic lymph node removal and numbers of lymph node removed with development of lymphedema.

To decrease the morbidity associated with complete lymphadenectomy, sentinel lymph node dissection is a novel method, being explored in vulvar cancers and other gynecological malignancies. Lymphedema in gynecology oncology patients manifests as leg, genital, or abdominal swelling after surgical removal of the lymph nodes, or pelvic RT for gynecologic or pelvic tumors. It generally occurs 1–2 years after treatment, but may occur even as late as 15 years. Lymphedema is a troublesome problem. Its early diagnosis is essential for its prevention. Many clinicians actually are not aware about its proper management. It may be due to recurrence of disease rather than the side-effects of cancer treatment. The patient’s morbidity is high and could actually be fatal. Lymphedema is the abnormal accumulation of lymph fluid under the skin and subcutaneous fatty tissue, leading to swelling of the limb, permanent skin changes, and cellulitis. Signs and symptoms of leg lymphedema are pain, swelling, reduced range of motion, muscle weakness, genital lymphedema, and difficulty with such activities of daily living as sitting or walking.[6] The diagnosis of lymphedema is one of exclusion. In addition to ruling out the recurrence, physicians must also rule out deep vein thrombosis as well as renal, liver, and heart disease. In this condition, swelling of the lower limb is not reduced with diuretics. Treatment consists of measures to reduce the swelling and to prevent cellulitis. It consists of acute intensive and maintenance phase. To decongest swelling, compression garment, manual lymph drainage, and exercise may help. After acute phase maintenance with continued skin care, remedial exercises and repeated self-massage is required. Aqualymphatic therapy is a novel approach done on a weekly basis, which is an effective, pleasurable, and inexpensive method to control lymphedema.[7] In vulva swelling, specially designed bandages with Velcro straps and odor control pads can be of use.

## DEEP VEIN THROMBOSIS

It causes significant morbidity and mortality. About 38% of gynecological oncology patients develop Deep Vein Thrombosis (DVT) in the post-operative period.[8] Pulmonary embolism causes a large number of deaths in high-risk patients for DVT in cervical and uterine cancer patients.[9] DVT prophylaxis with low-molecular weight heparin and pneumatic compression is helpful in the prevention of DVT in the post-operative period. Diagnosis is confirmed by Doppler study and treated with anticoagulants (conventional or low-molecular weight heparin followed by prolonged oral warfarin). Vena cava filters are used to prevent pulmonary emboli when, due to deranged coagulation profile, anticoagulants are contraindicated.

## SYMPTOMATIC ANEMIA

Because of chemotherapy, compromised hemopoisis in addition to hemorrhage and decreased oral intake may lead to severe anemia. Remedies are blood transfusions in addition to nutritional oral or parenteral support. Anemia due to hemopoitic disorder (post-chemotherapy or RT) needs proper evaluation and appropriate management with growth factors or fractionated red blood cells.

## BOWEL OBSTRUCTION

In patients with gynecological cancers, bowel obstruction can be partial or complete, and single or multiple. The clinical manifestations of bowel obstruction are nausea, vomiting, distension abdomen associated with pain, constipation, or overflow loose diarrhea. It is usually confirmed with abdominal radiographs demonstrating air fluid levels, but abdominal computed tomography (CT) scan defines the extent of disease hence helping in choosing therapeutic options of further antineoplastic therapy or surgery or only stenting, or pharmacological palliative intervention. The obstruction in gynecological malignancies could be due to various reasons and their management are also different.

Mechanical obstruction of the bowel is caused by occlusion of the lumen. Adynamic or paralytic intestinal obstruction is a functional obstruction. Infiltration of the mesentery or bowel muscles and nerves by tumor causes physiological obstruction. Other causes are inflammatory edema, fecal impaction, and constipating drugs. Sometimes, benign causes such as adhesions, post-radiation bowel damage, inflammatory bowel disease, or hernia may be the cause. Benign causes of obstruction are reported in about 6% of gynecological malignancies.[10]

Adynamic or paralytic bowel obstructions are relieved by conservative management. Isolated small bowel obstructions are treated by resection anastomosis, but multiple areas of partial small bowel obstruction are not amenable to surgical correction. This kind of small bowel obstruction is more challenging to manage. Tumor implants on the bowel surface and mesentery cause adhesions and impede peristalsis. A recent multidisciplinary working group of the European Association of Palliative Care reviewed issues regarding bowel obstruction and published clinical practice recommendations for the management of bowel obstruction in patients with end-stage cancer.[11] Palliative percutaneous gastrostomy tube draining by gravity can be performed or just suction with a nasogastric tube is possible. Somatostatin combined with erythromycin improves motility and decreases secretion to help in the management of this condition not amenable to surgical correction.

Surgery should not be routinely undertaken in patients with advanced and end-stage cancer who do not have a benign cause of occlusion, and will only benefit selected patients with mechanical obstruction and/or limited tumor, single site of obstruction, and those with a reasonable chance of further response to antineoplastic therapy. Before decision of surgery, the feasibility of relieving the obstruction and life expectancy after surgery should be assessed, and these are the major determinants of decision making. In patients with advanced ovarian cancer and no or minimal chance of response to antineoplastic treatment, for symptomatic relief of obstruction, decision should be taken jointly with the patient and family. Consent to palliative surgery should include discussion of the surgical risks, complications, and alternatives such as pharmacological management for symptom control.

## PAIN MANAGEMENT

Pain is the most common and most distressing symptom found in progressive gynecological malignancies.[12] In advanced or recurrent cervical cancer, pain could be due to involvement of the regional nerve, muscle, and bone. But, visceral pain could also be present as pelvic tumor encroaches the presacral area. In 60% cases, it is neuropathic due to involvement of the nerve trunk or sacrum.[13] Treatment depends on the type and pathophysiological mechanism. Pain should be managed with analgesics as per three steps (WHO analgesic ladder).[14] Palliative radiation has a role to relieve pain of bone metastasis or para-aortic lymph node recurrence. RT is the standard for palliation of painful bone metastases, aiding in effective pain relief and decreasing adverse effects from increased escalating opioid consumption.[15] Interventional techniques like epidural opioids and neurolysis_can be tried for patients with uncontrolled pain not responding to oral medications.

## PLEURAL EFFUSION

Malignant pleural effusion is a frequent complication of patients with advanced ovarian cancer, less frequently in endometrial or cervical cancer. The main aim in these patients is to relieve respiratory distress by removing the pleural fluid. Due to shortness of breath, the patient becomes anxious and opioids or benzodiazepines should be liberally used at end-of-life situation. In situations close to death with poor symptom control, a sedation therapy is required to palliate severe shortness of breath.[16,17] Intermittent drainage of pleural fluid is carried out for symptomatic relief, but repeated procedures result in pleural adhesions and result in failed tapping attempts. Continuous indwelling small catheter drainage is a relatively new technique. The patient can walk with the catheter, and outpatient management was shown to be feasible.[18] Pleurodesis with talc or bleomycin or tetracycline can be tried.

## RECURRENT ASCITES

Patients with advanced ovarian cancer and some with advanced endometrial cancer often need repeated drainage for malignant ascites.[19,20] Peritoneovenous shunts, paracentesis, and diuretics are available treatment options. In the late stage, paracentesis may be required weekly or more frequently as per patient comfort. In patients with no available effective anticancer therapy, a semi-permanent drainage tube can be placed for symptomatic relief. A Tenckhoff catheter can be placed for continuous drainage and the patient can manage it on her own, and multiple visits to the hospital could be avoided.[21] Repeated paracentesis of ascites leads to albumin depletion. Other adverse effects are infection, perforation, peritonitis, and hypotension. However, the immediate temporary improvement in patient comfort usually takes precedence over the long-term nutritional status for a patient who is terminally ill.

## CONSTIPATION

Pelvic recurrences can lead to partial or complete bowel obstruction, or it may be due to the side-effects of narcotic analgesics. Causes need to be explored and appropriately managed. Sometimes, simple measures like an increase in fluid intake or laxatives can be of benefit. More useful to the patient with cancer is the addition of fiber, colonic stimulants, and laxatives to their regimen.

## DIARRHEA

Radiation enteritis and proctitis are the most important causes of diarrhea in cases of gynecological malignancies. Increasing fluid intake and steroid enema help in symptomatic relief in these cases. Other agents that reduce diarrhea include anticholinergics and opiate derivatives such as loperamide, codeine, diphenoxylate sodium with atropine, Kaopectate, and cholestyramine.

## RECTOVAGINAL AND VESICOVAGINAL FISTULAE

Rectovaginal (RVF) and Vesicovaginal fistulae (VVF) can form as a result of locally progressive cervical cancer invading the bladder anteriorly or the rectum posteriorly. Progressive pelvic recurrences of ovarian cancer can also result in fistulaes. Rarely, VVF or RVF may be due to severe radiation proctitis without evidence of disease. Palliative diversion surgeries like permanent diverting colostomy or urinary diversion with uretero-ileal conduit are justified in cases where significant life expectancy is present or where disease is not present; but, in a terminally sick patient who is eating minimally, diapers with catheters with absorptive dressing may be helpful.[19]

## NAUSEA AND VOMITING

Uremia or bowel obstruction or metabolic imbalance may be responsible for nausea and vomiting. Proper evaluation and specific treatments are necessary for symptomatic improvement. Obstructive uropathy management has been discussed under the heading of uremia. Bowel obstruction can be corrected with resection and re-anastomosis, bowel bypass, ileostomy, percutaneous gastrostomy tube, or nasogastric tube. Colonic obstruction at the rectosigmoid requires transverse loop colostomy, or cecostomy. Hypercalcemia is an uncommon paraneoplastic manifestation of metastatic gynecologic cancer. Hydration, diuretics, steroids, calcium-binding agents, and bisphosphonates should be considered. For immediate symptomatic relief of nausea, phenothiazines antihistamines, steroids, or 5HT-3 antagonists can be used. Nausea and vomiting caused by brain metastases can be improved by the use of radiation therapy.

## PSYCHOSOCIAL ISSUES

Problems in India are definitely different than those in the western world. Although women literacy and financial independence is on a rise, still, a huge proportion of our women population are still ignorant, financially dependent, and illiterate. While they have limited decision-making powers and are under pressure to fulfil their other priorities in life, it has been noticed that they forget their own important health commitments easily. For early detection of recurrences and proper follow-ups, a government-based system, as a part of social welfare, is an urgent requirement to ensure long-term follow-ups. There has to be an area need-based modified system to ensure follow-ups and palliative care. Social support by providing conveyance and for children back at home, when a woman leaves the family for health needs, can be of help. Non-government social organisations or corporate organizations could contribute a lot. They can help in setting up and running cancer hospice centers for incurable malignancies if the mother is suffering from chronic illness like advanced ovarian malignancy or recurrent or residual progressive cervical cancer. In this scenario, young children can undergo an extreme kind of psychological adverse influence. In the Indian scenario, when the society is in transition from joint to nuclear type of family norm, even child nutrition and education can be jeopardized. Economic, psychological, spiritual, and other social needs, time to time assessment, and intervention prevent disintegration of family and indirectly help in continuity of treatment of patients with comparatively high morals, despite poor prognosis even in cases detected in advanced stages of cancer.

Psychosexual issues after treatment of gynecological cancers need to be addressed. Poor body image, depression, decreased interest in sex due to dryness and shortening of vagina, dyspaerunia, or due to fear of recurrence are commonly found in the post-treatment phase of gynecological cancer. Cancer of the uterus and cervix often involves treatment with pelvic RT. A permanent side-effect of RT is vaginal dryness, vascular and tissue damage, thinning of the epithelium, and atrophy of the vagina, with a reduction in elasticity and development of fibrosis.[22,23] About a third of women suffer from vaginal stenosis (obstruction by scar tissue) after pelvic RT.[22] Many of the women never resume normal sex life and suffer poor self-esteem and depression. Post-RT use of phallous-shaped vaginal dialator thrice weekly, each time for 5 min, may help in avoidance of adhesion formation in the vagina.

## QUALITY OF LIFE ISSUES

Similarly, the issues of dignity and quality of life in women with progressive incurable cancers and survivors of cancers need attention in our country. A women cured of disease may suffer debilitating side-effects like severe radiation proctitis, resulting in recto-vaginal fistula or obstructive uropathy. They need appropriate surgical management to improve their quality of life. Agony of a woman who suffers with continuously leaking urine or feces from a VVF or RVF, developed as a result of locally progressive cervical cancer, can be understood by no one except her. These women may get neglected in developing countries like India due to resource constraints, overburdened health systems, and lack of training of staff to deal with such kinds of issues. This requires strong and determined efforts by the health professionals and policy makers to develop sub-speciality-specialized courses in palliative care and hospice centers to let them have a peaceful last journey in dignity.

The aims of curative and palliative care are indeed the same; the enhancement of life with good quality of life. At different phases of disease, as time passes, readjustment of goals may be required. The patient and family members should never be left abandoned in the end at the time when all possible cancer control-targeted therapies are exhausted. This is a phase of extreme crisis and distress and needs sympathetic support and counselling to make proper decisions. In the absence of organized palliative care and hospice facilities in India, the burden of all of this falls on the head of the primary treating physician.
